# Diblock Copolymers Containing Titanium-Hybridized Polyhedral Oligomeric Silsesquioxane Used as a Macromolecular Flame Retardant for Epoxy Resin

**DOI:** 10.3390/polym14091708

**Published:** 2022-04-22

**Authors:** Ruirui Zhou, Lijie Lin, Birong Zeng, Xindan Yi, Chenyu Huang, Kunpeng Du, Xiaohui Liu, Yiting Xu, Conghui Yuan, Lizong Dai

**Affiliations:** 1 Department of Materials Science and Engineering, College of Materials, Xiamen University, Xiamen 361005, China; 20720181150077@stu.xmu.edu.cn (R.Z.); 20720191150057@stu.xmu.edu.cn (L.L.); 20720201150102@stu.xmu.edu.cn (X.Y.); libra20011015@163.com (C.H.); dkpeng0110@163.com (K.D.); 20720180154880@stu.xmu.edu.cn (X.L.); xyting@xmu.edu.cn (Y.X.); yuanch@xmu.edu.cn (C.Y.); 2 Fujian Provincial Key Laboratory of Fire Retardant Materials, Xiamen 361005, China

**Keywords:** Ti-POSS, phosphorus, macromolecular flame retardant, migration, epoxy resin

## Abstract

In this paper, the 9,10-dihydro-9-oxa-10-phosphaphenanthrene-10-oxide (DOPO)-containing diblock copolymer poly[(p-hydroxybenzaldehyde methacrylate)_m_-b-(2-((6-oxidodibenzo[c,e][1,2]oxaphosphinin-6-yl)oxy)ethyl methacrylate)_n_] (abbrev. poly(HAMA_m_-b-HEPOMA_n_)) was synthesized by reversible addition fragmentation chain transfer (RAFT) polymerization. When it was continued to react with titanium-hybridized aminopropyl-polyhedral oligomeric silsesquioxane (Ti-POSS) through a Schiff-base reaction, new grafted copolymers poly[(Ti-POSS-HAMA)_m_-b-HEPOMA_n_] (abbrev. PolyTi) were obtained. Then, they were used as macromolecular flame retardant to modify epoxy resin materials. The thermal, flame retardant and mechanical properties of the prepared EP/PolyTi composites were tested by TGA, DSC, LOI, UL-94, SEM, Raman, DMA, etc. The migration of phosphorus moiety from epoxy resin composites was analyzed by immersing the composites into ethanol/H_2_O solution and recording the extraction solution by UV-Vis spectroscopy. The results showed that the added PolyTi enhanced the glass transition temperature, the carbon residue, the graphitization of char, LOI, and mechanical properties of the EP/PolyTi composites when compared to pure cured EP. Furthermore, the phosphorus moieties were more likely to migrate from EP/DOPO composites than that from EP/PolyTi composites. Obviously, compared with small molecular flame retardant modified EP, the macromolecular flame retardant modified EP/PolyTi composites exhibited better thermal stability, flame retardancy, and resistance to migration.

## 1. Introduction

Epoxy resin (EP) is a very important polymer material, which has good thermal stability, mechanical properties and processability. However, epoxy resin is easy to burn and unsuitable for most applications. With more stringent requirements for environmental protection, flame retardant, and fireproof functions of materials, the development of flame-retardant modified epoxy resin composites have become an important research topic [1,2]. Usually flame retardant (FR) can be classified into several types, such as halogen FR [3], phosphorus FR [4], silicon FR [5], boron FR [6], nitrogen FR [7], sulfur FR [8], and metal compound FR [9]. For the sake of environmental friendliness and green environmental protection, many efforts have been focused to developing phosphorus- and silicon-containing compounds as new flame retardants, such as 9,10-dihydro-9-oxa-10-phosphaphenanthrene-10-oxide (DOPO) [10,11] and polyhedral oligomeric silsesquioxane (POSS) derivatives [12]. 

POSS is an organic-inorganic hybrid molecule with an inorganic Si-O skeleton as the cage core and the organic substituents at the eight corners as reactive functional groups. The general formula of POSS is (RSiO_1.5_)_n_ and the molecular size was about 1–3 nm, which is an intermediate between silica and polysiloxane (R_2_SiO)_n_. POSS can be incorporated into polymers by physical blending or copolymerization, endowing the composite materials with good mechanical and thermal properties. The related development of POSS has grown rapidly over the past few decades. For example, epoxy- or amino-functionalized POSS can participate in the epoxy curing process, thereby introducing POSS into the epoxy curing network. It is found that the POSS-modified epoxy resin has obvious self-extinguishing effect with a low heat release [13,14].

Due to the active P–H bond in the molecule, DOPO can react with other chemicals to generate a series of derivatives containing multiple flame retardant elements such as P-Si [15], P-N [16] and P-Si-N [17], which shows synergistic flame-retardant effects and are further used to improve the thermal stability and fire resistance of polymers. Recently, the combination of DOPO moieties and POSS units into one molecule has proved to be an efficient way to obtain new halogen-free flame retardant for epoxy resin composites [18]. 

Compared with the organic and inorganic flame retardants, metal compounds, including metal oxides [19], metal hydroxides [20], and polyoxometalate [21], are known to have outstanding advantages in catalyzing carbon formation, trapping free radicals, and suppressing fire smoke. At present, when metal oxides or metal hydroxides are used as flame retardants, a large addition amount about 20–40 wt% is required, which results in poor mechanical properties of polymer composites due to the poor compatibility of metal oxides/metal hydroxides/polyoxometalate with polymers. In order to improve the dispersion of metal in the polymer matrix, we have tried to incorporate metal elements (eg. Ti and Zr) into POSS to synthesize fully condensed metal-silsesquioxanes (M-POSS) at first, and then to combine it with other organic compounds such as DOPO through simple reactions to form a new flame retardant for achieving a high flame retardancy at a low addition amount less than 10 wt% [22,23,24,25].

Recently, there has been a growing concern about phosphorus flame retardant as emerging contaminants with increasing environment emission. For the sake of human health and environment safety, the release and exposure of phosphorus in water should be controlled. One strategy is to replace small organic phosphorus molecules such as organophosphate esters and DOPO derivates with phosphorus-containing macromolecules. In the present work, poly[(p-hydroxybenzaldehyde methacrylate)_m_-b-(2-((6-oxidodibenzo[c,e][1,2]oxa-phosphinin-6-yl)oxy)ethyl methacrylate)_n_] (abbrev. poly(HAMA_m_-b-HEPOMA_n_)) were synthesized by reversible addition fragmentation chain transfer (RAFT) polymerization. And then, titanium-hybridized aminopropyl-POSS (Ti-POSS) reacted with poly(HAMA_m_-b-HEPOMA_n_) via a Schiff-base reaction to synthesize grafted copolymers poly[(Ti-POSS-HAMA)_m_-b-HEPOMA_n_] (abbrev. PolyTi), which contained DOPO and Ti-POSS units. Meanwhile, the grafted copolymer without titanium element poly[(POSS-HAMA)_m_-b-HEPOMA_n_] (abbrev. PolyC) was also synthesized from poly(HAMA_m_-b-HEPOMA_n_) and aminopropyl POSS for a comparison. The chemical structure and thermal properties of the polymers were characterized by ^1^H NMR, FITR, XRD, TGA, and DSC. In addition, the POSS-grafted and Ti-POSS-grafted copolymers were added to epoxy resin as flame retardants to prepare the EP/PolyC and EP/PolyTi composites, respectively. Finally, the thermal, flame retardant and mechanical performance of the prepared epoxy resin composites were tested by TGA, DSC, LOI, UL-94, SEM, Raman, DMA, etc., and the influence of a block ratio with a different Ti content on the flame retardancy was discussed. Moreover, the migration of phosphorus moiety DOPO from epoxy resin composites was evaluated by immersing the composites into ethanol/H_2_O solution and recording the extraction solution by UV-Vis spectroscopy.

## 2. Materials and Methods

### 2.1. Materials

P-hydroxybenzaldehyde (98%), methacryloylchloride (98%), triethylamine (99%), dichloromethane (DCM, 99.5%), hydroxyethyl methacrylate (96%), azodiisobutyronitrile (AIBN, 98%), tetrahydrofuran (99%), methanol (99.5%), DOPO (97%) and 4,4’-diaminodiphenylmethane (DDM, 99%) were purchased from Aladdin (Shanghai, China). Magnesium sulfate anhydrous (99%), carbon tetrachloride (99.5%) and acetonitrile (99%) were purchased from Sinopharm Chemical Reagent Co. Ltd., China. 4-cyano-4-dithiobenzoate valeric acid (CPAD, 97%) was purchased from Merck (Darmstadt, Germany). Aminopropyl polyhedral oligomeric silsesquioxane (NH_2_-POSS, 98%) was supplied by Hybrid Plastics Company (Hattiesburg, MS, USA). Isopropyl titanate (98%) and tetraethyl ammonium hydroxide (40% in H_2_O) were supplied by J&K Scientific LTD (Beijing, China). All chemicals were used as received. Ti-hybridized aminopropyl-POSS (Ti-POSS) was prepared in the lab according to the previous report [22,23,24,25].

### 2.2. Synthesis of DOPO-Containing Copolymers and Ti-POSS Grafted Copolymers

**Scheme 1:**
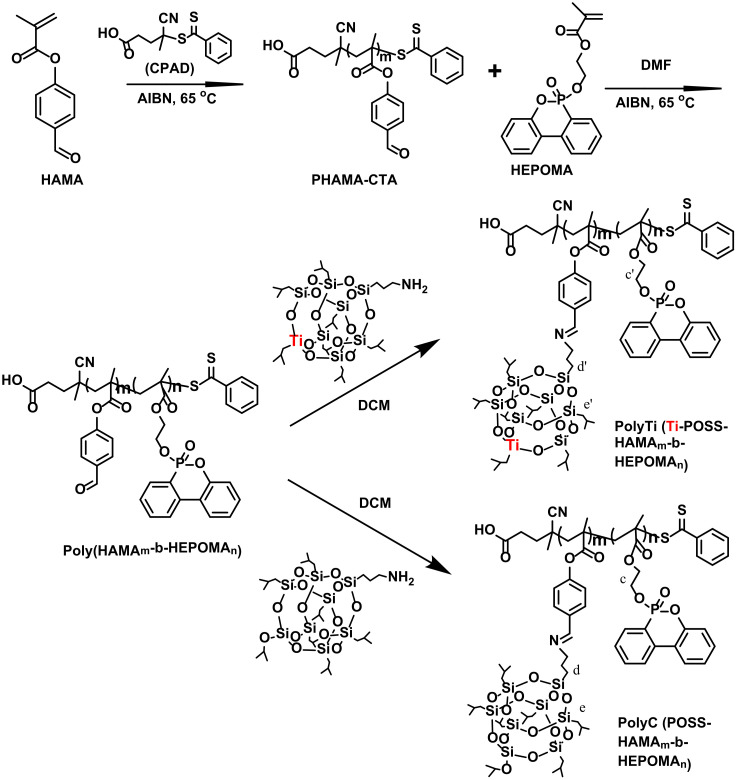
Synthetic route of poly(HAMA_m_-b-HEPOMA_n_), poly[(Ti-POSS-HAMA)_m_-b-HEPOMA_n_] (PolyTi), and poly[(POSS-HAMA)_m_-b-HEPOMA_n_] (PolyC).

As shown in Scheme 1, the monomers p-hydroxybenzaldehyde methacrylate (HAMA) and 2-((6-oxidodibenzo[c,e][1,2]oxaphosphinin-6-yl)oxy)ethyl methacrylate (HEPOMA) could be obtained according to the previous work [26,27]. The DOPO-containing copolymers poly(HAMA_m_-b-HEPOMA_n_) with different block ratios were synthesized by RAFT polymerization. Firstly, 4 g (21.03 mmol) of HAMA, 0.1952 g (0.700 mmol) of CPAD, 0.0230 g of (0.133 mmol) AIBN and 8 mL of tetrahydrofuran were added to a 25 mL Schlenk reaction tube. After freeze-thawing with liquid nitrogen, the reaction tube was placed in a water bath at 65 °C for 24 h with the protection of argon. The macro-chain transfer agent PHAMA-CTA was collected after filtration, washed with cold methanol and dried in a vacuum. Secondly, PHAMA-CTA (0.5783 g, 0.0967 mmol), HEPOMA (0.5000 g, 1.45 mmol) and AIBN (0.0003 g, 0.0019 mmol) were dissolved into DMF (10 mL) in a Schlenk reaction tube. After freeze-thawing with liquid nitrogen, the mixture continued to react at 65 °C for 24 h. By filtration, washing with THF and drying in vacuum, the DOPO-containing block copolymer poly(HAMA_28_-b-HEPOMA_16_) was collected. According to stoichiometric calculation and adjusted the feeding weight of HEPOMA monomer, three DOPO-containing copolymers with different block ratios were obtained. They were poly(HAMA_28_-b-HEPOMA_16_), poly(HAMA_28_-b-HEPOMA_30_) and poly(HAMA_28_-b-HEPOMA_109_), which were named as Poly1, Poly2 and Poly3.

The synthesis of Ti-POSS grafted copolymers: firstly, Poly1 (1.1160 g), Poly2 (1.6320 g) and Poly3 (4.7280 g) separately dissolved in DCM under stirring. Secondly, Ti-hybridized aminopropyl-POSS (Ti-POSS, 2.688 g, 3 mmol) were added into the Poly1, Poly2 and Poly3 solution, respectively. After reacting for 12 h at room temperature, the mixture was concentrated with a rotary evaporator and then precipitated with 200 mL acetonitrile. The Ti-POSS-grafted copolymers of poly[(Ti-POSS-HAMA)_28_-b-HEPOMA_16_], poly[(Ti-POSS-HAMA)_28_-b-HEPOMA_30_] and poly[(Ti-POSS-HAMA)_28_-b-HEPOMA_109_] were obtained, which were named as PolyTi1, PolyTi2 and PolyTi3. 

Similarly, added the stoichiometric aminopropyl POSS (NH_2_-POSS, 2.6240 g, 3 mmol) into Poly2/DCM solution. After reacting at room temperature for 12 h, the target product of POSS-grafted copolymer poly[(POSS-HAMA)_28_-b-HEPOMA_30_] was obtained by filtration. It was named as PolyC and used as a comparison sample.

### 2.3. Preparation of EP/Polyti Composites

PolyTi was added into DGEBA (E51) for preparing EP/PolyTi composites. Firstly, E51 was heated to 60 °C and stirred for 20 min. Secondly, PolyTi was dissolved in DCM, and then dropped into E51. After stirring for 30 min, it was heated up to 90 °C and a certain amount of DDM was added. Thirdly, after all the PolyTi and DDM was mixed homogeneously, a vacuum pump was used to remove the solvent, water, air and other small molecules. Finally, the mixture was poured into an aluminum mould and thermally cured at 120 °C for 4 h, 140 °C for 2 h and 180 °C for 2 h. The compositions of the prepared EP/PolyTi composites were listed in Table 1. Using the same curing procedure, the pure EP was prepared by a stoichiometric amount of DGEBA and DDM. Meanwhile the cured EP/DOPO was achieved by feeding stoichiometric amounts of DGEBA, DDM and DOPO/Ti-POSS blend.

### 2.4. The Migration of DOPO Moiety from EP Composites

A series of approximately 1.0 g EP composite samples with similar total surface area were weighed and then put into tubes. A total of 10 mL of 10% ethanol solution and 50% ethanol solution were added into tubes, respectively. Then, the samples were sealed and kept at room temperature for 8 months. Finally, the extraction solution was removed and the absorption curve was recorded by UV-Vis spectroscopy.

### 2.5. Characterization

^1^H NMR spectra were recorded on a Bruker Avance 400 MHz NMR spectrometer (BRUKER, Berlin, Germany) with a standard 5 mm probe at 25 °C using CDCl_3_ as solvent. IR spectra were characterized by a Nicolet iS10 FTIR (Thermo Fisher, Waltham, MA, USA). XRD spectra were tested by Bruker-AXS XRD (D8-A25) (BRUKER, Billerica, MA, USA) with a scan angle of 5–80°. Thermogravimetric analysis (TGA) was tested on a Netzsch STA 409EP by heating from 35 to 800 °C at a heating rate of 10 °C/min under air or inert gas atmosphere. DSC was measured by using Netzsch STA 449C heating from room temperature to 250 °C under N_2_. The limited oxygen index (LOI) was conducted on fire testing technology oxygen index with samples size 80 × 10 × 4 mm according to ISO 4589. The vertical burning test was performed by using a FTT 0082 UL-94 instrument according to IEC 60695-11-10. Scanning electron microscopy (SEM) was conducted on an SU-70 microscope to observe the morphology of the inner and outer surfaces of the residual carbon and the morphological structures of the fracture surfaces. The release of DOPO moiety was determined by a UV-2550 spectrometer (SHIMADZU, Kyoto, Japan).

## 3. Results and Discussions

### 3.1. Chemical Structural Characterizations

#### 3.1.1. DOPO-Containing Copolymers poly(HAMA_m_-b-HEPOMA_n_)

**Figure 1:**
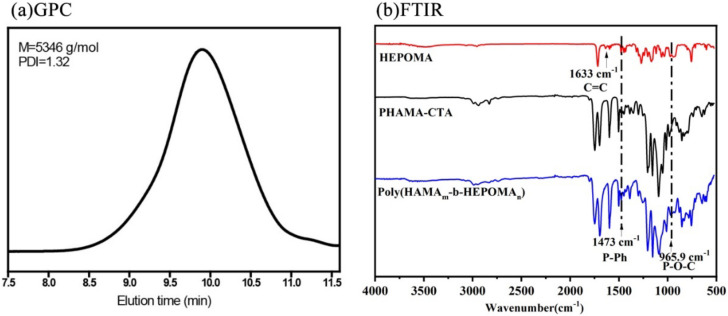
(**a**) The GPC of HAMA-CTA, (**b**) FTIR spectra of HEPOMA, PHAMA-CTA and poly(HAMA_m_-b-HEPOMA_n_).

The GPC curve of PHAMA-CTA macromolecular chain transfer agent (Figure 1a) presented that the molecular weight of PHAMA-CTA was about 5346 g/mol with a PDI of 1.32. It could be calculated that the polymerization degree of PHAMA-CTA was about 28. The IR spectra of HEPOMA monomer, PHAMA-CTA and poly(HAMA_m_-b-HEPOMA_n_) were shown in Figure 1b. It could be seen that HEPOMA monomer had -C=C, P-Ph and P-O-C bonds, which located at 1633 cm^−1^, 1473 cm^−1^ and 965.9 cm^−1^, respectively [28]. However, in the IR spectrum of poly(HAMA_m_-b-HEPOMA_n_), the C=C bond peak at 1633 cm^−1^ disappeared, while those of the P-Ph bond and P-O-C bond remained. This proved that the copolymer poly(HAMA_m_-b-HEPOMA_n_) was successfully synthesized.

**Figure 2:**
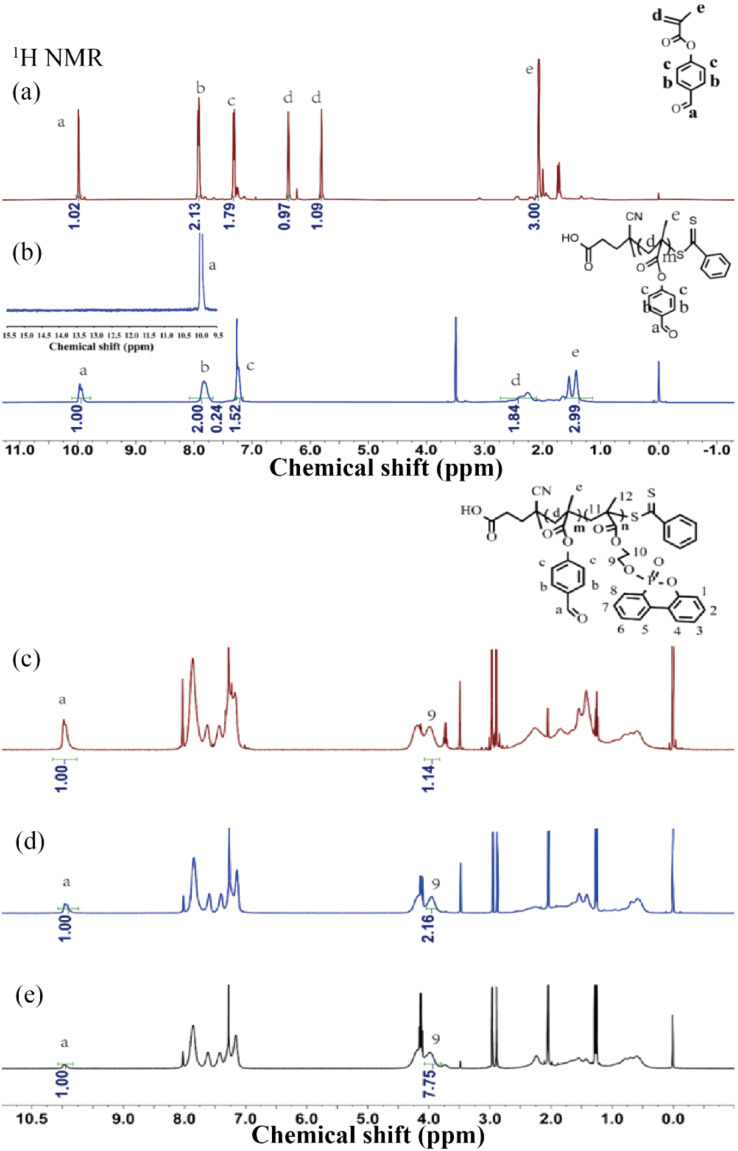
The ^1^H NMR spectra of (**a**) HAMA, (**b**) HAMA-CTA, (**c**–**e**) poly(HAMA_m_-b-HEPOMA_n_).

The ^1^H NMR of HAMA, PHAMA-CTA and poly(HAMA_m_-b-HEPOMA_m_) were shown in Figure 2. Figure 2a showed that HAMA had -CH_3_, CH_2_=C-, -CHO and benzene protons, which located at 2.06 ppm, 6.35 and 5.78 ppm, 9.96 ppm, 7.84 and 7.24 ppm, respectively [26]. It was seen in Figure 2b that the characteristic peak at 6.35 and 5.78 ppm disappeared, meanwhile, the new peaks of methylene (2.10–2.74 ppm) and methyl (1.31–1.61 ppm) appeared, which indicated that HAMA monomers polymerized to produce PHAMA-CTA. Figure 2c–e showed the ^1^H NMR spectra of poly(HAMA_m_-b-HEPOMA_n_) with different block ratios. The integration areas of the aldehyde proton H_a_ (δ 9.98 ppm) and the methylene proton H_9_ (δ 4.28 ppm) could be calculated and the ratio value represented the polymerization degree ratio of HAMA-containing block and HEPOMA-containing block. The calculation results (Table 2) showed that the synthesized poly(HAMA_m_-b-HEPOMA_n_) were poly(HAMA_28_-b-HEPOMA_16_), poly(HAMA_28_-b-HEPOMA_30_) and poly(HAMA_28_-b-HEPOMA_109_), respectively.

#### 3.1.2. Grafted Copolymers PolyTi and PolyC

**Figure 3:**
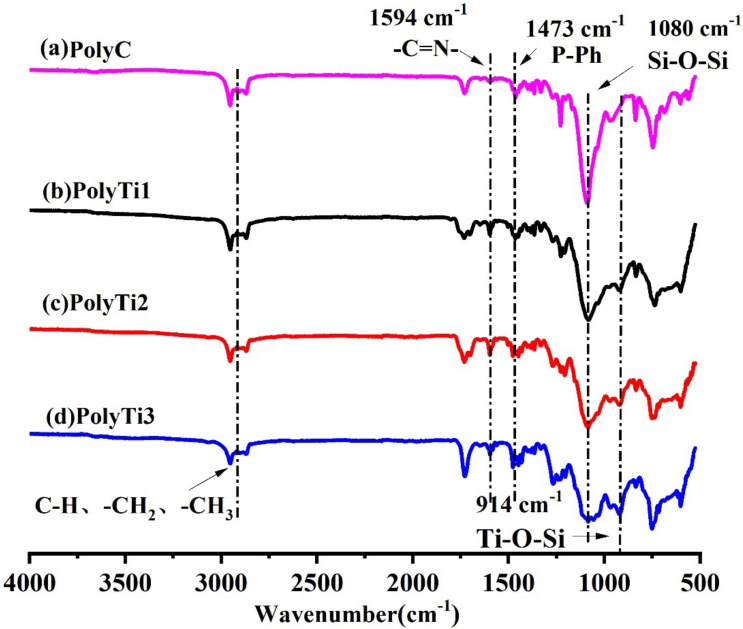
FTIR spectra of (**a**) PolyC, (**b**) PolyTi1, (**c**) PolyTi2 and (**d**) PolyTi3.

The aminopropyl POSS and Ti-hybridized aminopropyl POSS molecule could be further grafted to poly(HAMA_m_-b-HEPOMA_n_) via a Schiff-base condensation reaction to synthesize poly[(POSS-HAMA)_28_-b-HEPOMA_30_] (PolyC) and poly[(Ti-POSS-HAMA)_m_-b-HEPOMA_n_] (PolyTi). The IR spectra of PolyC and PolyTi were shown in Figure 3. Compared with poly(HAMA_m_-b-HEPOMA_n_) in Figure 2, it could be found that two new absorption peaks at 1594 and 1080 cm^−1^ appeared, which indicated the formation of C=N bond and Si-O-Si bond, respectively. In addition, there also appeared a new peak at 914 cm^−1^ for PolyTi, which was assigned to the stretching vibration absorption of Ti-O-Si [22]. The IR results proved the successful synthesis of POSS or Ti-POSS grafted copolymers PolyC and PolyTi. 

**Figure 4:**
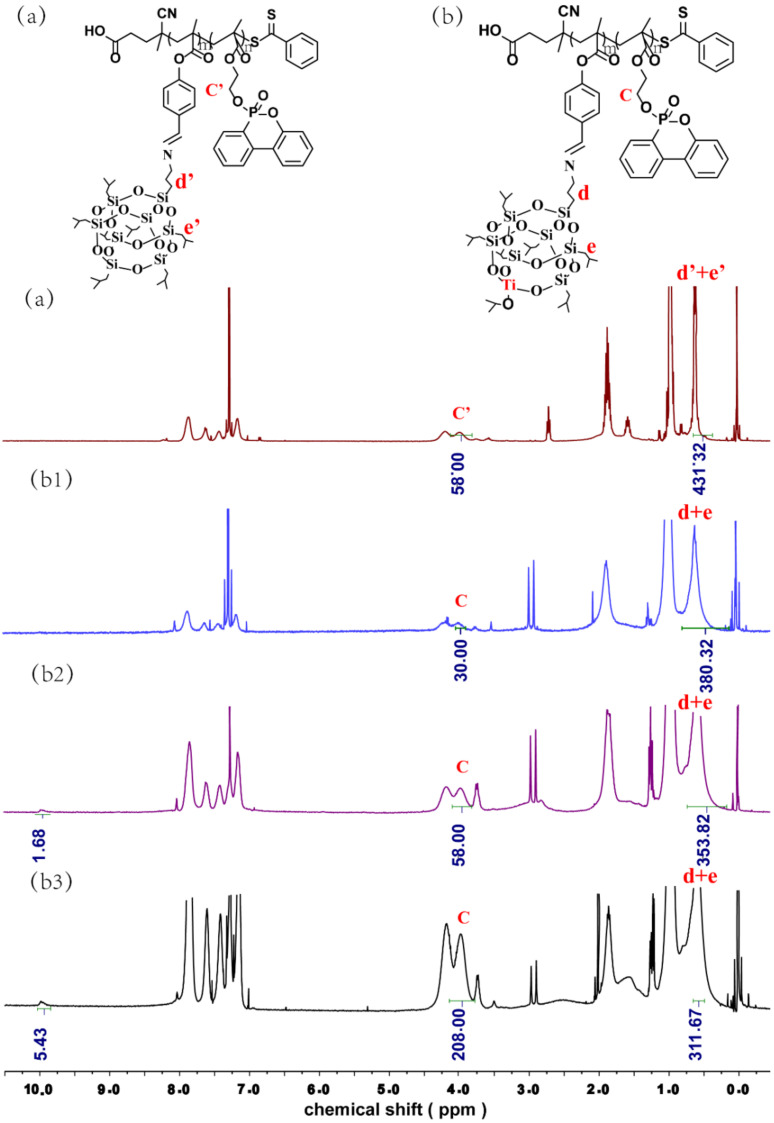
The ^1^H NMR spectra of grafted polymer: (**a**) PolyC, (**b1**–**b3**) PolyTi1, PolyTi2, and PolyTi3.

The NMR spectra of PolyC and PolyTi were given in Figure 4. Compared with poly(HAMA_m_-b-HEPOMA_n_) (Figure 2c–e), new characteristic peaks corresponding to -Si-CH_2_-CH(CH_3_)_2_ appeared in PolyC. Meanwhile, both the peaks corresponding to -Si-CH_2_-CH(CH_3_)_2_ and Ti-O-CH(CH_3_)_2_ were observed in PolyTi. The integration areas were well consistent with the theoretical proton number. Therefore, it also verified that PolyC and PolyTi were successfully synthesized.

### 3.2. Thermal Stability of Grafted Copolymers PolyTi and PolyC

#### 3.2.1. Thermal Properties of Grafted Copolymers

**Figure 5:**
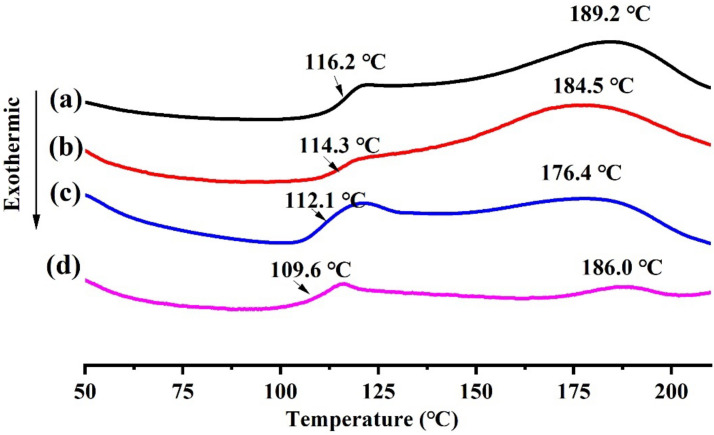
The DSC curves of the (**a**) PolyTi1, (**b**) PolyTi2, (**c**) PolyTi3 and (**d**) PolyC.

DSC and TGA were applied to investigate the thermal stability of materials. It was seen from Figure 5 that the glass transition temperature T_g_ of PolyTi1, PolyTi2, PolyTi3 and PolyC were 116.2 °C, 114.3 °C, 112.1 °C and 109.6 °C, respectively. Obviously, all the T_g_ of PolyTi were higher than that of PolyC. Additionally, with the decreasing in the block ratio m/n, the T_g_ of the corresponding copolymer showed a slight downward trend; that is, the lower the relative content of Ti-POSS-containing block, the lower the T_g_ of its corresponding copolymer. This might be explained by the reason that the volume and rigidity of Ti-POSS octahedral cage structure were greater than that of HEPOMA units, which made the movement of the Ti-POSS-containing block chain more hindered. The polymerization degree of HEPOMA-containing block in PolyTi1, PolyTi2 and PolyTi3 was 16, 30 and 109, respectively. When the relative proportion of Ti-POSS-containing segments in PolyTi was smaller, it made the movement of molecular segments easier, and then decreased the T_g_, so as to obtain the T_g_ trend to be PolyTi1 > PolyTi2 > PolyTi3. The DSC curves also showed that PolyC and PolyTi had obvious melting endothermic peaks in the high-temperature region. The melting temperature of PolyTi1, PolyTi2 and PolyTi3 was 198.2 °C, 184.5 °C and 176.4 °C, respectively. It was explained that the cage-like POSS provided the physical cross-linking points, which increased the interaction between the molecular chains and affected the melting temperature of PolyTi. As the polymerization degree of the HEPOMA-containing block turned larger, the relative proportion of Ti-POSS was diluted and became smaller, thereby weakening the forced interactions between the overall molecular chains of PolyTi. Therefore, the melting temperature tended to decrease from PolyTi1 to PolyTi3. In addition, PolyTi2 and PolyC had almost the same block ratio. Here, the melting temperature of PolyTi2 was slightly lower than that of PolyC (186.0 °C). The reason might be that the presence of Ti in Ti-POSS units broke the symmetry of the cage structure as the physical crosslinking points, resulting in a decrease in the interaction force between molecular chains, to a certain extent.

#### 3.2.2. Thermal Stability of Grafted Copolymers

**Figure 6:**
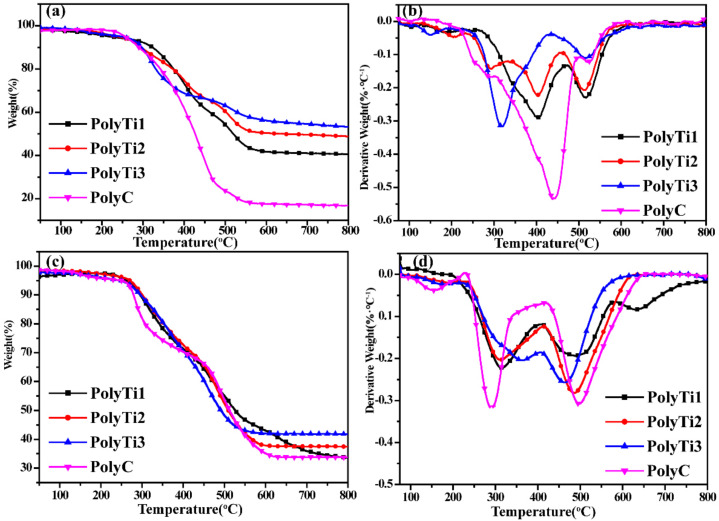
TGA and DTG curves of PolyTi and PolyC: (**a**,**b**) in N_2_; (**c**,**d**) in air.

Figure 6 showed the TGA and DTG curves of PolyTi and PolyC. The initial decomposition temperature (T_d_) is 5% mass loss temperature. T_max1_ and T_max2_ correspond to the temperature of two main thermal weight loss respectively. As shown in Figure 6a, under nitrogen atmosphere, the T_d_ of PolyC and PolyTi2 were 255.8 °C and 238.9 °C respectively, and the T_max1_ were 442.0 °C and 404.1 °C, respectively. Apparently, both T_d_ and T_max1_ of PolyC were higher than those of PolyTi2, which might be due to the lower thermal degradation temperature of Ti-O-C bond than Si-C bond, and similar phenomena had been reported in other literatures [29,30]. By analyzing the DTG curve in Figure 6b, it was found that compared with PolyC, the maximum thermal weight loss rate of PolyTi2 was relatively reduced by 0.317 wt%·min^−1^. This suggested that the introduction of Ti into the polymer could reduce the decomposition rate of grafted polymer. Additionally, the carbon residue rate of PolyTi2 was increased. It was seen that at 800 °C, the residual carbon of PolyC and PolyTi was 16.79 wt% and 48.76 wt% respectively. In addition, the T_d_ of PolyTi1, PolyTi2 and PolyTi3 were 218.9 °C, 238.9 °C and 245.9 °C, respectively, indicating that the initial thermal stability of the polymers gradually increased with the extension of the HEPOMA-containing block. The T_max1_ of PolyTi1, PolyTi2 and PolyTi3 were 402.3 °C, 404.1 °C and 318.3 °C respectively. The T_max_ of PolyTi3 was found to be significantly reduced. This might be because the proportion of Ti-POSS-containing block decreased significantly with the increase of the polymerization degree of the HEPOMA block to reach 109, which meant that the heat-resistance effect of the Ti-POSS-containing chain decreased. The heat resistance of Ti-POSS was derived from the Si-C bond (292 kcal/mol) and Si-O bond (452 kcal/mol) with large bond energy in the POSS framework structure [31]. The copolymer containing POSS structure first degraded the organic group part of the shell of POSS, and then degraded the cage-like structure core to form a silicon-containing carbon layer, which improved the thermal stability of the copolymer. Therefore, a smaller proportion of Ti-containing POSS block affected the maximum thermogravimetric temperature and maximum thermogravimetric rate of the polymer. The initial maximum thermogravimetric temperature and maximum thermogravimetric rate of PolyTi2 were slightly higher than those of PolyTi1, which was caused by the contribution of HEPOMA block. Considering the degree of polymerization and thermal stability, PolyTi2 had the best effect, and m/n block ratio was close to 1:1. Figure 6c,d showed the TG and DTG curves of the four grafted copolymers in air atmosphere. Due to the presence of oxygen, the oxidative decomposition was more thorough and the weight loss was also greater than those under N_2_. However, the decomposition trend was the same as that under an N_2_ atmosphere. The decomposition was mainly due to the breaking of C-C, C-P, and P-O-C bonds, resulting in the initial weight loss, which in turn led to the degradation of the POSS structure.

#### 3.2.3. XRD Analysis

**Figure 7:**
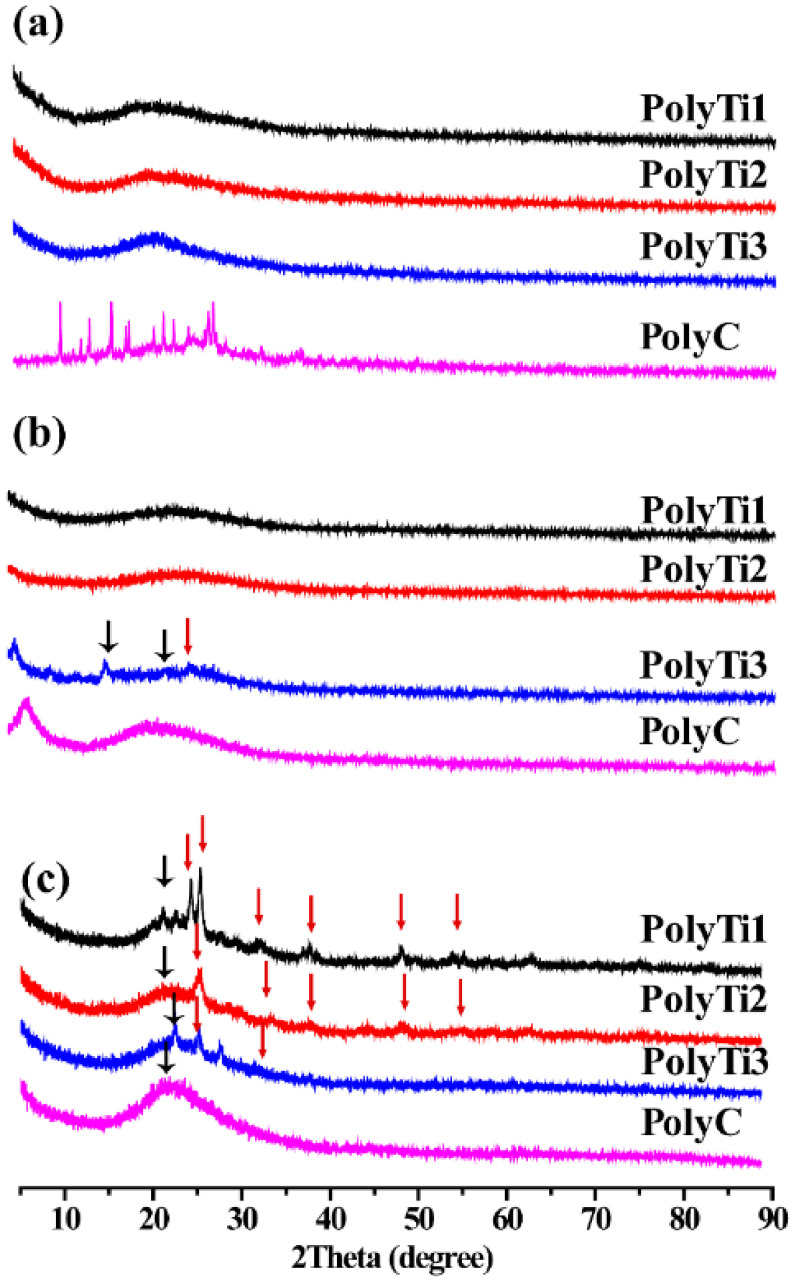
XRD spectra of PolyTi and PolyC: (**a**) unsintered, (**b**) sintered residue at 400 °C, and (**c**) sintered residue at 800 °C.

In order to further explore the degradation of PolyC and PolyTi, the sintered products were characterized by XRD at 400 °C and 800 °C, respectively, and the results were shown in Figure 7. From the XRD spectra in Figure 7a, it was seen that the peaks of PolyTi looked like broad bump, showing an amorphous state. However, PolyC had some clear crystal phase peaks. It was speculated that the Ti-hybridized POSS had an influence on the crystal form because the introduction of Ti might affect the symmetry of the POSS cage. Decomposition occurred due to the sintering of the polymer at high temperatures. It was seen in Figure 7b that after sintering at 400 °C, three characteristic peaks appeared at 15.7° (022), 21.9° (030) and 25.4° (110) in the XRD spectrum of PolyTi3, which was assigned to the ingredient of SiO_2_ [32] and TiO_2_ [22] in the residual carbon. As shown in Figure 7c, when the sintering temperature reached 800 °C, the characteristic SiO_2_ crystallization peak at about 21.9° (030) appeared in each block polymer. Meanwhile, the characteristic TiO_2_ crystallization peaks [33,34] at 25.4° (110) and 31.7° (400), 37.1° (202), 48.0° (020), and 53.5° (601) could be found in the carbon layer of the three PolyTi.

### 3.3. The Compatibility of PolyTi and PolyC in Epoxy Resin

**Figure 8:**
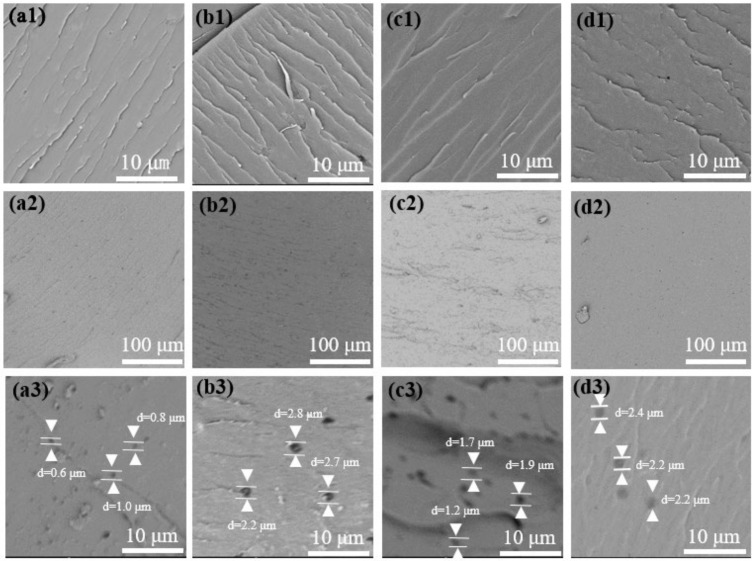
SEM section images of (**a1**–**a3**) EP/PolyTi1, (**b1**–**b3**) EP/PolyTi2, (**c1**–**c3**) EP/PolyTi3, and (**d1**–**d3**) EP/PolyC before and after etching.

Usually, DOPO molecules dissolve well in epoxy resin, while POSS is not easy to disperse. Therefore, it is necessary to analyze the compatibility of PolyTi and PolyC with epoxy resin when used as flame retardants. Figure 8 showed the SEM of cured epoxy resin mixed with PolyTi and PolyC before and after etching by dichloromethane as a good solvent. No large grains were observed on the surface of the EP/PolyTi and EP/PolyC composites, indicating that both PolyTi and PolyC were uniformly dispersed in the epoxy resin. After the EP composites were placed in dichloromethane, the flame retardants could be extracted and some holes appeared on the surface. The etched pore size of EP/PolyTi1, EP/PolyTi2, EP/PolyTi3 and EP/PolyC were about 0.8 μm, 2.5 μm, 1.6 μm, and 2.2 μm, respectively, which indicated that PolyTi and PolyC formed self-assembled aggregates because Ti/POSS-containing blocks and HEPOMA-containing blocks had different affinities to epoxy resins. 

### 3.4. Flame Retardancy of EP Composites

The LOI and UL-94 tests of EP composites, the Raman and SEM of residual carbon were characterized. The results (Table 3) showed that the LOI value of EP was 23.0%. Compared with EP, the LOI values of EP/PolyTi1, EP/PolyTi2, EP/PolyTi3 and EP/PolyC were improved by 18%, 25%, 23% and 22%, respectively. All EP/PolyTi composites passed the UL-94 V0 grade. It was inferred that the addition of PolyTi could effectively enhance the flame retardancy of EP. When the block ratio of Ti-POSS-HAMA to HEPOMA block was close to 1:1, the LOI of PolyTi2 could be effectively enhanced. In addition, the block ratios of PolyTi2 and PolyC were almost the same, but the LOI of PolyTi was higher than that of PolyC, indicating that Ti element promoted the flame retardancy of POSS and DOPO for EP composites. 

**Figure 9:**
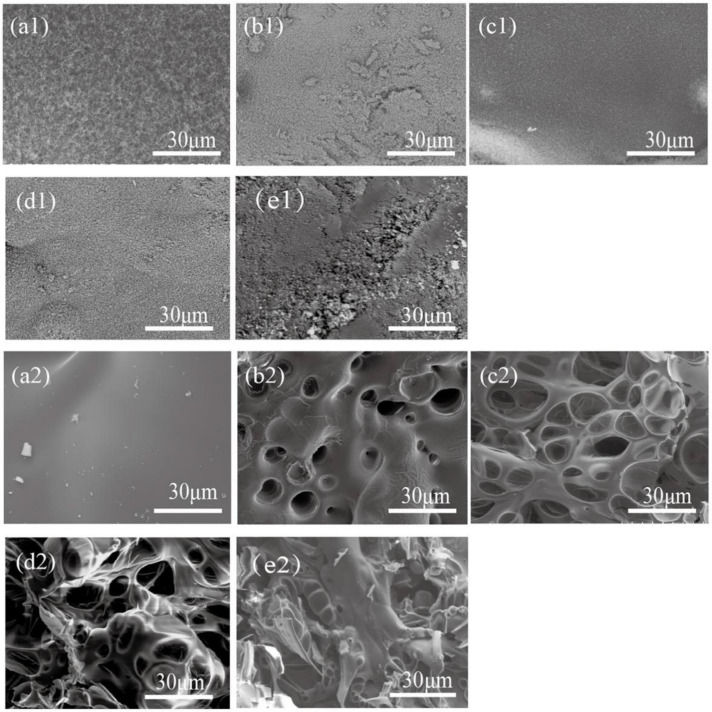
SEM of carbon residue: (**a1**,**a2**) EP, (**b1**,**b2**) EP/PolyTi1, (**c1**,**c2**) EP/PolyTi2, (**d1**,**d2**) EP/PolyTi3, (**e1**,**e2**) EP/PolyC. Outer layer: (**a1**–**e1)** and inner layer: (**a2**–**e2**).

The SEM characterization of the carbon residues in EP composites were shown in Figure 9. It was seen that the outer residues of the EP composites (Figure 9a1–e1) were fluffier and rougher than the inner residues (Figure 9a2–e2). During the addition of PolyTi to EP, the carbon residue in the outer layer of the EP composites was denser and more continuous than pure EP, which acted as a physical barrier and was beneficial to reduce the penetration of the oxygen and ambient heat. At the same time, it could be observed that the inner layer of the residual carbon in the EP composites exhibited a honeycomb-like morphology and had many visible bubbles (Figure 9b2–d2), which meant that the melt viscosity of the composites increased after the addition of PolyTi, making more gas fixed in the EP matrix. Therefore, PolyTi slowed down the ablation of the inner matrix. As PolyC was added to the epoxy resin, the residual carbon of EP/PolyC composite was found to be better than that of pure EP, but worse than that of EP/PolyTi, which proved that the existence of Ti element was helpful to enhance the flame retardancy.

**Figure 10:**
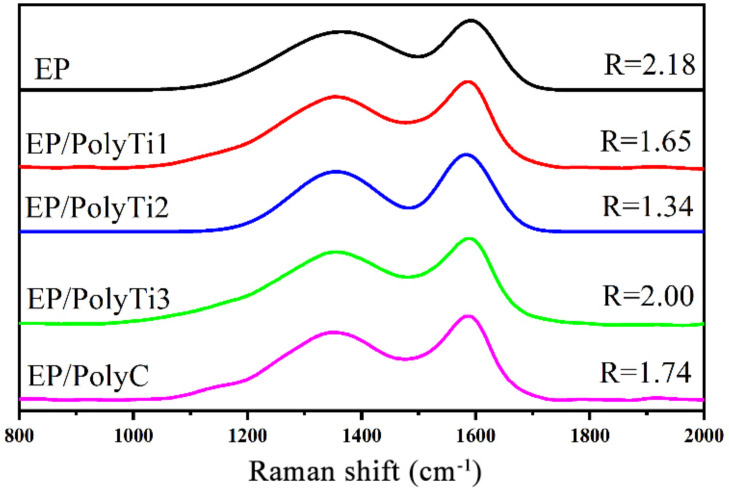
The Raman spectra of carbon residue of EP composites.

Raman is an important means of analyzing carbon materials. Figure 10 showed the Raman spectra of the EP composite’s char. There had two characteristic absorption peaks around 1355 cm^−1^ (D peak) and 1613 cm^−1^ (G peak), which were assigned to disordered carbon and ordered carbon, respectively [22]. The ratio ID/IG (designated R) of the peak intensities of the D and G was used to measure the degree of graphitization of the carbon residue. The more ordered carbon formed, the lower the R, representing a higher degree of graphitization. It could be seen from Figure 10 that the R value of the carbon residue after adding PolyTi was lower than that of pure epoxy resin (R = 2.18), indicating that the addition of PolyTi could improve the degree of graphitization of the carbon layer and make it easier to form a dense carbon layer. The order of R values for the three EP/PolyTi composites was EP/PolyTi1 (1.34) < EP/PolyTi2 (1.65) < EP/PolyTi3 (2.00). Among them, the R value of EP/PolyTi2 was the smallest, indicating that it was most favorable for the formation of ordered carbon. Obviously, the block ratio (m/n) had an influence on the formation of ordered carbon. When the ratio of the Ti-POSS-containing block to DOPO-containing block was close to 1, the interaction between phosphorus, silicon and titanium could reach an optimized degree to obtain the most ordered carbon, thereby resulting in a more stable carbon layer. In addition, the R value of the EP/PolyC was 1.74, which was slightly higher than that of EP/PolyTi2, indicating that the presence of Ti element in the flame retardant was beneficial to improve the degree of graphitization of carbon residues.

### 3.5. Thermal Properties of EP Composites

**Figure 11:**
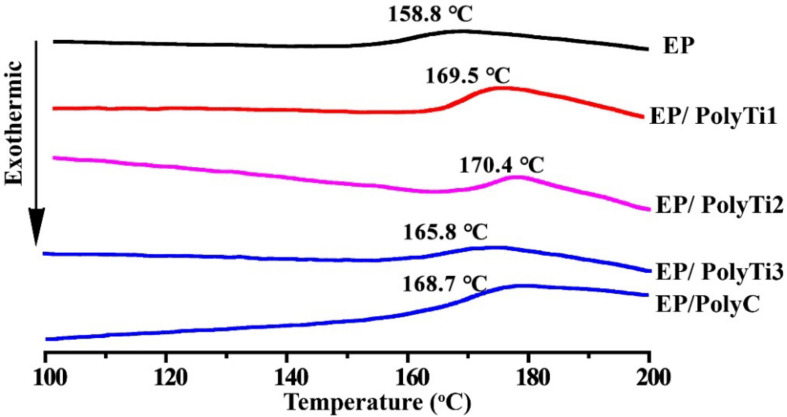
The DSC curves of EP/PolyTi and EP/PolyC composites.

As shown in the DSC curve in Figure 11, there was only one T_g_ peak in the EP/PolyTi cured system, indicating that when PolyTi was added to EP, they had good compatibility and became homogeneous after mixing. The T_g_ of pure cured EP was 158.8 °C, and the T_g_ of EP/PolyTi1, EP/PolyTi2 and EP/PolyTi3 were 169.5 °C, 170.4 °C and 165.8 °C, respectively. Compared with pure cured EP, the addition of PolyTi increased T_g_ by 6.7%, 7.3%, 4.4%, respectively. It was inferred that the rigid cage-like structure of Ti-POSS in the Ti-POSS-HAMA block provided a bulk steric effect that reduced the motion of macromolecular chains, leading to elevated T_g_. At the same time, the DOPO-containing block also had a volume steric effect due to the rigid benzene rings in the molecules. Therefore, the combination of Ti-POSS-containing block and DOPO-containing block was expected to promote T_g_. If the block ratio (m/n) of Ti-POSS to DOPO was increased from 28/16 to 28/30, the two rigid structural impediments of a Si-O cage and a benzene ring were added together, thus making the T_g_ of EP/PolyTi2 be greater than that of EP/PolyTi1. However, it was worth noting that the DOPO-containing block mainly contributed to the solubility of PolyTi, whereas the Ti-POSS-containing block did not. As the block ratio (m/n) of Ti-POSS to DOPO increased continuously from 28/30 to 28/109, the hindering effect of POSS would be diluted, and the macromolecular chain was stretched well due to the improved dispersion, which in turn reduced the T_g_ of EP/PolyTi3. It confirmed that the block ratio (m/n) had an influence on thermal properties of the EP/PolyTi composites. In addition, the T_g_ of the EP/PolyC composite sample without Ti hybridization was 168.7 °C, which was slightly lower than that of EP/PolyTi2. This indicated that the presence of Ti element in the flame retardant was beneficial to improve the T_g_ of EP composites. 

**Figure 12:**
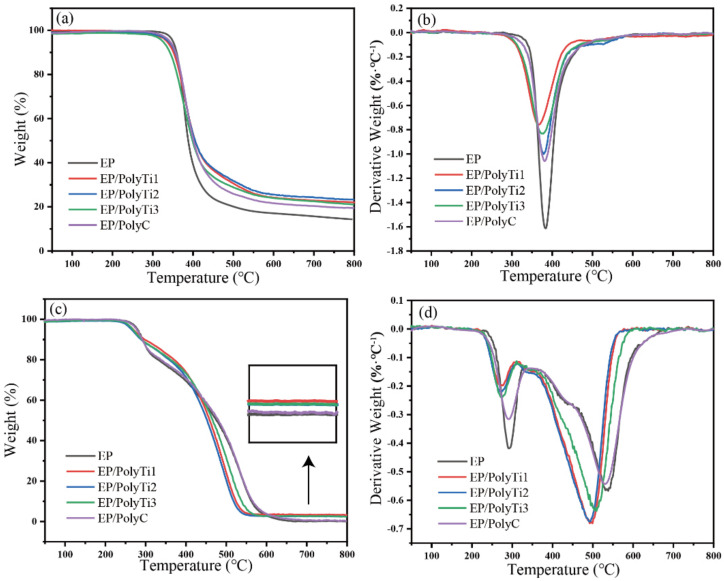
TGA results of the epoxy composite materials in N_2_ (**a**,**b**) and air (**c**,**d**) atmospheres.

To further explore the effect of PolyTi on the thermal properties of the modified EP composites, TGA tests were carried out in nitrogen and air atmospheres, respectively. The results were shown in Figure 12, and the relevant data are listed in Table 4. In a N_2_ atmosphere, the decomposition process of EP composites was completed in one stage, while in air atmosphere, the decomposition of EP composites included two stages. The results showed that under nitrogen atmosphere, the carbon residue value of EP at 800 °C was 14.3%, while those of EP/PolyTi1, EP/PolyTi2, and EP/PolyTi3 were 22.0%, 23.3% and 21.1%, respectively. Compared with pure EP, all the carbon residue values were increased. When the ratio of the Ti-POSS-containing block to the DOPO-containing block was close to 1, the carbon residue value of EP/PolyTi2 reached its maximum. It was speculated that there was a synergistic effect between P, Si and Ti, thereby improving the thermal stability of the EP composites. From the DTG curve (Figure 12b), it could be seen that under N_2_, the maximum thermal degradation rate after adding PolyTi (<1.0 wt%∙°C^−1^∙min^−1^) was much lower than that of EP (1.6 wt%∙°C^−1^∙min^−1^), indicating that PolyTi could slow down the thermal degradation process of epoxy resin. This was because the Ti-POSS unit with rigid cage structure and Si-O-Si bonds with higher bond energy as well as the high flame retardant efficiency of DOPO contributed to the thermal stability of EP/PolyTi composites. Additionally, the EP/PolyC without Ti formed 19.5 wt% carbon residue at 800 °C, accompanying with a decomposition rate of near 1.1 wt%∙°C^−1^∙min^−1^, which was between the thermal performance of EP and EP/PolyTi. What’s more, in air atmosphere (Figure 12c,d) the T_d_ of EP/PolyTi1, EP/PolyTi2, EP/PolyTi3 and EP/PolyC were 268.3 °C, 264.8 °C, 263.0 °C and 277.5 °C, respectively. At 800 °C, their carbon residue was 3.24%, 2.40%, 2.44% and 0.19%, respectively. This indicated that although the breakage of the P-O-C/P-C bonds in DOPO occurred at the early stage [25], which reduced T_d_, the formed polyphosphates conversely promoted the carbonization of EP composites and inhibited the combustion degradation process. In a word, the thermal stability of EP composites was improved under the joint effect of Ti-POSS moieties and DOPO units.

### 3.6. Mechanical and Thermomechanical Properties of EP Composites

**Figure 13:**
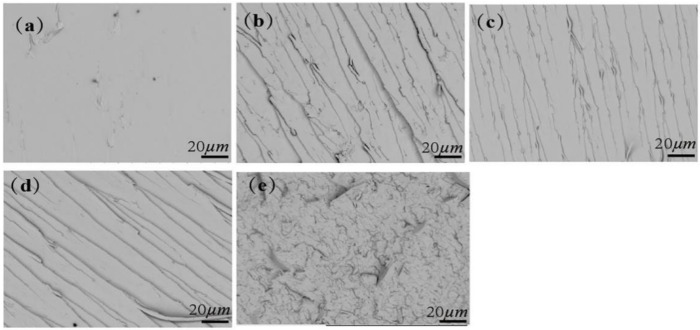
SEM images of fracture surfaces of (**a**) EP, (**b**) EP/PolyTi1, (**c**) EP/PolyTi2, (**d**) EP/PolyTi3, and (**e**) EP/PolyC.

EP composites were broken in liquid nitrogen for the observation of the fracture surfaces by SEM. The SEM images of all the EP composites were shown in Figure 13. It was seen that the fracture surface of EP was smooth (Figure 13a), indicating that it was brittle fracture for pure EP. When PolyTi was added to EP, as shown in Figure 13b–d, the fracture surface of EP/TolyTi composites became rough with some stripe textures, presenting a typical ductile fracture. In addition, as shown in Figure 13e, the cross-section of EP/PolyC maintained a rough surface, but the streaked texture was less pronounced than that of EP/PolyTi. In order to clarify the influence of the flame retardant on the mechanical properties of the EP composites, the dynamic thermomechanical properties were studied by a three-point bending test and DMA.

**Figure 14:**
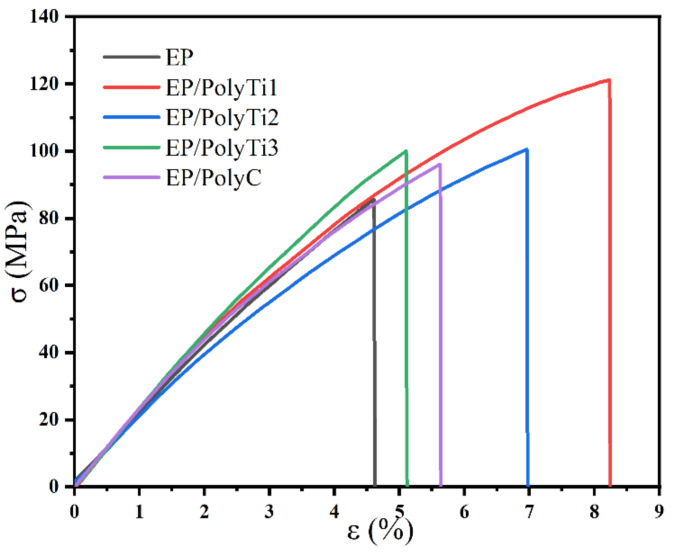
Stress–strain curves of the EP composites.

The stress–strain curve of the EP composites was shown in Figure 14, and the relevant data of three-point bending test were shown in Table 5. The maximum bending strength and fracture energy of the cured EP was 178.32 MPa and 394.33 N/m, respectively. When the flame retardant of PolyTi or PolyC was added to epoxy resin, the bending strength and fracture energy of the EP composites were higher than those of EP. Obviously, the cage-like structure of Ti-POSS increased the free volume between chain segments, and the macromolecular chains were prone to displacement after being stressed, which played a role of transferring energy. Therefore, the mechanical properties of the modified EP composites were improved. In addition, the flexural strength of EP/PolyC was lower than that of EP/PolyTi2, indicating that the effect of Ti-POSS-containing block on the mechanical properties of epoxy resin was better than that of POSS-containing block.

**Figure 15:**
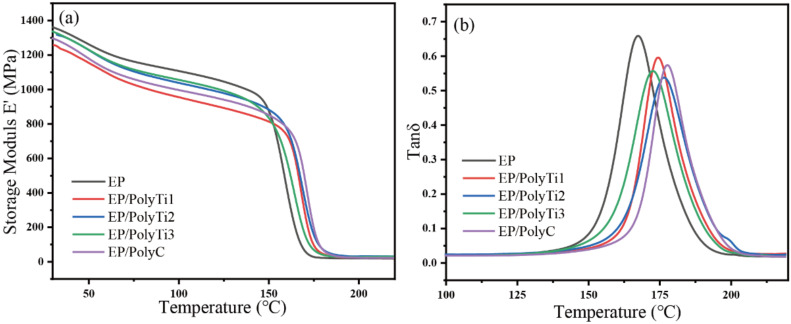
DMA curves of the EP composites: (**a**) Storage Moduls E′ and (**b**) Tanδ.

The DMA curves of the EP composites were shown in Figure 15. The results showed that the glassy storage modulus E′-40 at 40 °C was 1315, 1211, 1288, 1287, and 1245 MPa for EP, EP/PolyTi1, EP/PolyTi2, EP/PolyTi3, and EP/PolyC, respectively. Meanwhile, the rubber storage modulus E′-190 at 190 °C was 20.3, 26.0, 35.8, 27.8, and 28.0 MPa for EP, EP/PolyTi1, EP/PolyTi2, EP/PolyTi3, and EP/PolyC, respectively. Since the flame retardants of PolyTi and PolyC were non-reactive polymeric additives, they usually could not enter the epoxy cross-linked network obtained by the reaction of the curing agent and epoxy molecules. On the one hand, when PolyTi or PolyC was added to epoxy resin, the macromolecular flame retardants might dilute the crosslink density produced from the curing agent. On the other hand, they could self-assemble into aggregates to provide some physical cross-linking points. If the physical crosslinking enhancement effect of PolyTi or PolyC was stronger than the dilution effect, the overall crosslinking density would increase. This explanation was consistent with the experimental results of the tanδ value. As shown in Figure 15, the tanδ values of EP, EP/PolyTi1, EP/PolyTi2, EP/PolyTi3, and EP/PolyC were 0.66, 0.60, 0.54, 0.56, and 0.57, respectively, indicating that the crosslinking density of EP was the smallest. Therefore, the addition of PolyTi or PolyC could improve the mechanical properties of EP composites.

### 3.7. The Migration of DOPO from EP Composites

**Figure 16:**
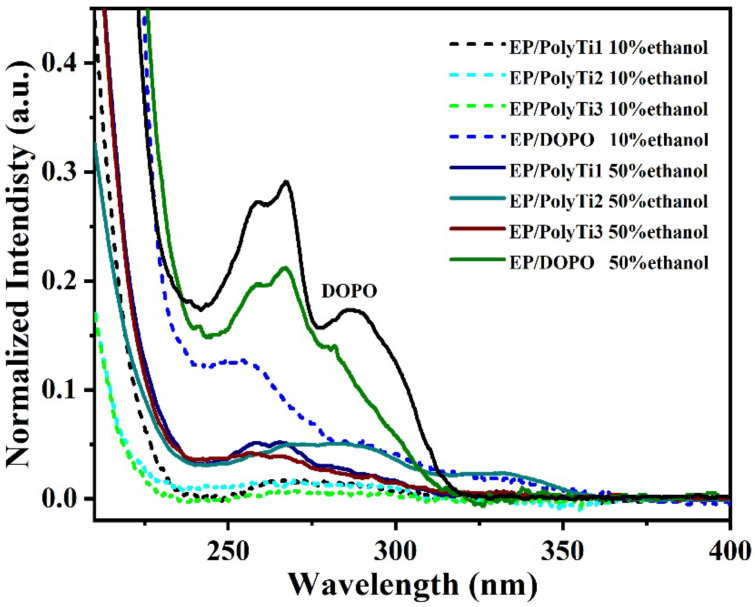
The UV-vis absorption spectra of the extraction solution of EP/PolyTi1 and EP/DOPO in 10% ethanol (dash line) and in 50% ethanol (solid line).

In the experiments, EP composites were immersed in 10% ethanol solution and 50% ethanol solution for 8 months, respectively. The extraction solution was characterized by UV-vis spectroscopy. Figure 16 illustrated the UV-vis spectra of the ethanol solution of DOPO, EP/PolyTi and EP/DOPO. The UV-vis spectrum of DOPO exhibited strong absorption peaks at 260 nm, 270 nm and 285 nm [35]. The UV-vis spectrum of the extraction solution of EP/DOPO also appeared the corresponding peaks of DOPO, which meant that it occurred the migration of DOPO from EP composites into ethanol aqueous solution. Additionally, the migration was more serious when immersing in a 50% ethanol solution than in a 10% ethanol solution. That is, organic solvent would increase the migration of the flame retardant. However, almost no signal was observed for all the EP/PolyTi composites in 10% ethanol, implying that PolyTi did not release from EP/PolyTi composites even if immersing for 8 months. In 50% ethanol, although a tiny release of DOPO moiety could be observed for the EP/PolyTi composites, it was still much weaker than EP/DOPO. It could be concluded that compared with small molecular phosphorus flame retardant, the macromolecular flame retardant PolyTi exhibited a better resistance to migration and less release into the environment, which might be more beneficial to environment protection.

### 3.8. Comparison of Properties of Obtained Material with Materials Described in the Literature

Furthermore, we compared the EP/PolyTi composites prepared in this study with previously published related flame retardant epoxy resin systems containing the components of POSS and DOPO as well as Ti. The comparative data were listed in Table 6. Among them, the EP/(5%Ti-POSS-5%DOPO) composite was prepared by adding 5 wt% Ti-POSS and 5 wt% DOPO blended mixture [23]. The EP/6% Ti-POSS-bisDOPO composite was prepared by adding 6 wt% Ti-POSS-bisDOPO compound [22]. The EP/5%POSS-bisDOPO-5%Titanate composite was prepared by adding 5 wt% POSS-bisDOPO and 5% isopropyl titanate. The results showed under the combination system of POSS, DOPO and titanium element, the EP composites could be well modified to pass the UL-94 V0 rating. The comparison results also showed that the macromolecular flame retardant PolyTi could produce the similar flame retardancy as the other three flame retardant system. What is more, it could effectively improve the bending strength of EP composites. Another special advantage of PolyTi was that it could increase T_g_ to a relatively high temperature, which was expected to meet the working requirement of high temperature conditions, thereby expanding its application prospects. 

## 4. Conclusions

In this paper, three diblock copolymers with different degrees of polymerization, poly(HAMA_28_-b-HEPOMA_16_), poly(HAMA_28_-b-HEPOMA_30_) and poly(HAMA_28_-b-HEPOMA_109_), were synthesized by RAFT polymerization. Then, the diblock copolymer flame retardants of poly[(Ti-POSS-HAMA)_m_-b-HEPOMA_n_] containing Ti-POSS moiety and DOPO units were prepared by a aldehyde amine condensation Schiff-base reaction. It showed that the cage-like structure of POSS in side-chains provided physical cross-linking points, which improved the interaction between polymer chains and made the melting temperature higher. The cage structure of Ti-POSS primitive can increase the free volume between chain segments, and the molecules are prone to displacement after being stressed, which played a role in transmitting energy. Therefore, the higher the content of Ti-POSS primitive in a certain range, the better the mechanical properties. When the ratio m/n of the two blocks in PolyTi was about 1, the EP/PolyTi2 composite could achieve good comprehensive properties. Compared with pure EP, the T_g_ of EP/PolyTi2 was 170.4 °C and the LOI was 28.8%, which was increased by 7.3% and 25.0%, respectively. In addition, the maximum bending strength and fracture energy of the cured EP was 178.32 MPa and 394.33 N/m, respectively. Meanwhile, the storage modulus of EP/PolyTi2 composite was 1288 MPa, higher than that of EP/PolyC with a value of 1245 MPa. Obviously, the addition of PolyTi could raise T_g_ of EP/PolyTi composites to a relatively high temperature to meet the working requirements under a high temperature. What is more, the phosphorus moieties were less likely to migrate from EP/PolyTi composites than that from EP/DOPO. In a word, the new macromolecular flame retardant PolyTi can play an important role in improving the degree of graphitization of char, flame retardancy, thermal and mechanical properties of the modified EP/PolyTi composites, accompanying with a better resistance to migration, which will be of significance to application aspect and environment protection.

## Figures and Tables

**Table 1:** The compositions of the EP/PolyTi composite materials.

Samples	DGEBA+DDM (wt%)	Poly[(Ti-POSS-HAMA)_m_-b-HEPOMA_n_]	Element Content (%)		
			P	Si	Ti
EP	100	0	0	0	0
EP/PolyC	95	5	0.21	0.77	0
EP/PolyTi1	95	5	0.12	0.77	0.20
EP/PolyTi2	95	5	0.22	0.68	0.17
EP/PolyTi3	95	5	0.50	0.40	0.10

**Table 2:** The block ratios of Poly(HAMA_m_-b-HEPOMA_n_) calculated by ^1^H NMR.

Sample	Integration Area		The Molar Ratio of HAMA: HEPOMA m/n = 2A_a_/A_9_	Chemical Formula of Poly(HAMA_m_-b-HEPOMA_n_)
	Peak H_a_ -CHO/ δ 9.98	Peak H_9_ -CH_2_-O-/ δ 4.28		
Poly1	1.00	1.14	1:0.57	poly(HAMA_28_-b-HEPOMA_16_)
Poly2	1.00	2.16	1:1.08	poly(HAMA_28_-b-HEPOMA_30_)
Poly3	1.00	7.75	1:3.9	poly(HAMA_28_-b-HEPOMA_109_)

**Table 3:** The LOI value and UL-94 test of the EP composites with 5 wt% addition.

Samples	LOI (%)	UL-94			
		Burning Grade	T1 (s)	T2 (s)	Dripping
EP	23.0	NR	>30	/	Yes
EP/PolyTi1	27.1	V-0	1	5	No
EP/PolyTi2	28.8	V-0	1	7	No
EP/PolyTi3	28.2	V-0	1	5	No
EP/PolyC	28.1	V-0	7	5	No

**Table 4:** The TGA data of the EP composites.

Samples	Nitrogen			Air			
	T_d_/°C	T_max_/°C	Char/wt%	T_d_/°C	T_max1_/°C	T_max2_/°C	Char/wt%
EP	349.9	383.2	14.3	279.4	291.1	537.5	0.02
EP/PolyTi1	334.3	367.4	22.0	268.3	270.2	496.3	3.24
EP/PolyTi2	338.2	380.5	23.3	264.8	272.0	492.2	2.40
EP/PolyTi3	324.6	376.0	21.1	263.0	273.0	506.6	2.44
EP/PolyC	342.3	380.5	19.5	277.5	289.9	523.6	0.19

**Table 5:** The data of three-point bending of EP composites.

Sample	δ_max_ (MPa)	Fracture Energy (N/m)
EP	178.32	394.33
EP/PolyTi1	249.85	987.47
EP/PolyTi2	209.37	699.54
EP/PolyTi3	208.35	510.39
EP/PolyC	200.14	540.29

**Table 6:** The comparison of several flame retardant system for the modification of epoxy resin.

Sample	LOI	Increase Rate (%)	UL-94	DSC T_g_	Increase Rate (%)	Char (wt% in Air)	Bending Strength (MPa)	Increase Rate (%)	Resource
EP/5%PolyTi2	28.8	25.0	V-0	170.4	7.3	2.4	100.7	18.4	This work
EP/5%Ti-POSS+5%DOPO	32.7	29.8	V-0	158.5	0.6	1.3	90.51	27.1	[23]
EP/6%Ti-POSS-bisDOPO	39.2	56.8	V-0	163.8	7.7	2.1	86.12	11.1	[22]
EP/5%POSS-bisDOPO+5%Titanate	31.0	18.3	V-0	161.3	4.0	1.3	_	_	[36]

## Data Availability

Data are available in a publicly accessible repository.
